# Curation-free biomodules mechanisms in prostate cancer predict recurrent disease

**DOI:** 10.1186/1755-8794-6-S2-S4

**Published:** 2013-05-07

**Authors:** James L Chen, Alexander Hsu, Xinan Yang, Jianrong Li, Younghee Lee, Gurunadh Parinandi, Haiquan Li, Yves A Lussier

**Affiliations:** 1 Ctr for Biomed Informatics and Dept of Medicine, The University of Chicago, Chicago, IL, USA; 2 Depts of Biomedical Informatics and Internal Medicine, Ohio State University College of Medicine, Columbus, OH, USA; 3 Depts of Medicine & of Bioengineering, University of Illinois at Chicago, Chicago, IL, USA; 4 University of Illinois Hospital and Health Science System, Chicago, IL, USA; 5 University of Illinois Cancer Center, Chicago, IL, USA

## Abstract

**Motivation:**

Gene expression-based prostate cancer gene signatures of poor prognosis are hampered by lack of gene feature reproducibility and a lack of understandability of their function. Molecular pathway-level mechanisms are intrinsically more stable and more robust than an individual gene. The Functional Analysis of Individual Microarray Expression (FAIME) we developed allows distinctive sample-level pathway measurements with utility for correlation with continuous phenotypes (e.g. survival). Further, we and others have previously demonstrated that pathway-level classifiers can be as accurate as gene-level classifiers using curated genesets that may implicitly comprise ascertainment biases (e.g. KEGG, GO). Here, we hypothesized that transformation of individual prostate cancer patient gene expression to pathway-level mechanisms derived from automated high throughput analyses of genomic datasets may also permit personalized pathway analysis and improve prognosis of recurrent disease.

**Results:**

Via FAIME, three independent prostate gene expression arrays with both normal and tumor samples were transformed into two distinct types of molecular pathway mechanisms: (i) the curated Gene Ontology (GO) and (ii) dynamic expression activity networks of cancer (Cancer Modules). FAIME-derived mechanisms for tumorigenesis were then identified and compared. Curated GO and computationally generated "Cancer Module" mechanisms overlap significantly and are enriched for known oncogenic deregulations and highlight potential areas of investigation. We further show in two independent datasets that these pathway-level tumorigenesis mechanisms can identify men who are more likely to develop recurrent prostate cancer (log-rank_p = 0.019).

**Conclusion:**

Curation-free biomodules classification derived from congruent gene expression activation breaks from the paradigm of recapitulating the known curated pathway mechanism universe.

## Background

Over the past decade, a plethora of genomic prostate cancer signatures have proliferated. A simple PubMed search reveals over 20,000 entries for genomic signatures ranging from traditional mRNA, miRNA, and SNP arrays to whole-exome sequencing. Despite this wealth of signatures and the fact that prostate cancer remains the second most common cancer among US men, not a single prostate cancer gene signature is available for commercial use. But why this clinical disconnect? Others and we have identified the lack of stability, interpretability, and personalization of these genomic signatures [1,2] as key impediments to their more widespread adoption.

At its simplest, genomic signatures are merely statistically significant differences between dichotomized phenotypes. Yet these phenotypes are heterogeneous and in prostate cancer have demonstrated low predictive power of mRNA based genomic signatures [3]. Even with well-matched, coherent phenotypes, the specter of underpowered statistics due to corrections for multiplicity remains a very real problem [4].

Fortunately, pathway-level mechanisms offer an elegant solution in enhancing the power and understandability of these genomic signatures. For the sake of clarity, in this article we use the terms "molecular pathway" and "molecular mechanism" to describe both conceptual and bioinformatically derived aggregations of genes such as in the Gene Ontology as well as protein-protein interaction subnetworks as referenced above. Work by the Ideker lab has repeatedly demonstrated the stability of protein network-based signatures over conventional differentially expressed genes [5]. In other words, perturbations between two phenotypes lie at the network level and not at any one genomic marker. Indeed in prostate cancer, we have demonstrated the conservation of molecular pathways among multiple prostate gene signatures of poor prognosis [1].

Nevertheless, the use of pathway-based mechanisms has been hampered by the difficulty (i) in generating pathway signatures for an individual patient and (ii) in unbiasedly and systematically evaluating molecular pathways. Traditionally, biologists have conducted a number of experiments to develop gene sets associated with specific pathways; however these approaches are rate-limiting when attempting to scale up to a large number of pathways [6]. Further, previously developed computational approaches to generate mechanism-anchored gene expression classifiers either failed to produce accurate classifiers as in the case of straightforward median or mean-based gene expression [7], or require discrete group assignments of multiple patients in their learning algorithm [5,8]. While the latter are exceptionally useful for better understanding conserved mechanisms of disease among populations of patients, it does not tell us what is true for an individual patient. Importantly, to our knowledge, all these algorithms testing multiple mechanisms were exclusively validated using human curated and manually annotated gene sets (e.g. Gene Ontology).

Ideally then, we would like to leverage the stability of pathway-level genomics at the level of an individual patient. The Functional Analysis of Individual Microarray Expression [9], was developed to address these issues and was used to evaluate head and neck cancer datasets using curated and pathway annotated. FAIME computes mechanism scores using rank-weighted gene expression of an individual sample. Each sample comprises its "profile of mechanisms", which allows for correlations with continuous variables such as survival time. In that regard, FAIME differs substantially from state of the art algorithms (e.g. GSEA [10]) that identify mechanisms across samples rather than within each sample, and are not designed for correlations.

In this extension of the FAIME algorithm, we hypothesized that personalized mechanism profiling using FAIME could be applied to unbiased, curation-free computationally generated mechanisms (Cancer Modules). Cancer Modules may provide the ability to detect pathways that do not have a defined pathway in the literature and thus are not beholden to existing domain knowledge. We further hypothesized that they would be predictive of clinical recurrence, which remains a hard problem for clinicians treating men with prostate cancer. We validate this hypothesis in the prostate cancer domain, for which no mechanism-anchored signatures have been reported.

## Methods

### Dataset sources and pre-processing

To determine whether an individualized pathway-level mechanisms were indeed superior to standard differentially expressed genes, we examined three publically available independent datasets: Yu [11], Wallace [12], and Taylor [13] to derive signatures of prostate tumorigenesis. We then examined an additional dataset to evaluate the prognostic significance with regard to survival [14] dataset. Table 1 describes these datasets in detail. The Yu, Taylor and Wallace datasets were directly downloaded from the normalized form deposited in GEO. The Glinsky dataset [14] was obtained from the author.

**Table 1:** Prostate cancer datasets analyzed

Author	Phenotype	Samples Analyzed	Usage	Source
[11]	Normal vs Tumor	Authors examined 61 microdissected prostate cancer specimens along with 63 normal prostate tissues samples adjacent to tumor of patients.	Feature Selection	GDS2547
[12]	Normal vs Tumor	Authors examined 33 African-American and 36 European-American patients with prostate cancer. Also profiled 18 non-tumor prostate tissues from 7 African-American and 11 European-American patients.	Feature Selection	GSE6956
[13]	Normal vs Tumor	Comprehensive set from Memorial Sloan Ketting tumor bank in prostate tumors from 53 patients with primary or metastatic prostate cancer and 29 normal controls.	Feature Selection	GSE21032
[14]	Disease free interval survival	79 tumors were obtained from the Memorial Sloan Kettering tumor bank. The authors identified three signatures of early recurrence (within one year).	Validation Set	From author

**Figure 1:**
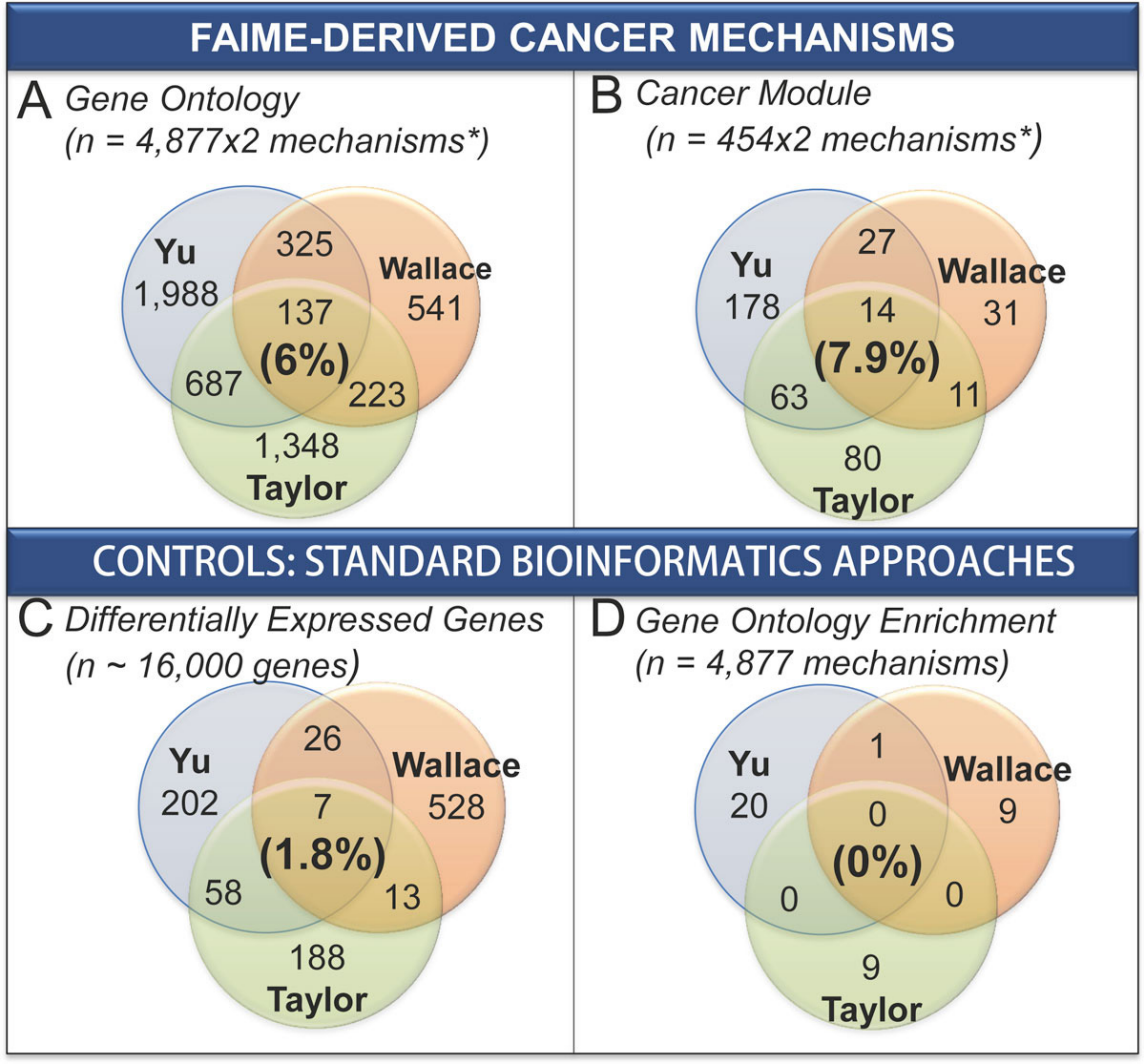
**FAIME transformation of gene expression arrays into Gene Ontology space (Panel A) and Cancer Module Space **(**Panel B) improves overlap by three to four fold as compared to standard bioinformatics techniques as demonstrated by differential gene expression (Panel C) or Gene Ontology enrichment of the differentially expressed genes (Panel D)**. In this analysis, we compared 3 different prostate cancer datasets (Yu, Wallace, Taylor) for differences between tumor and normal prostate gene expression in 4 different analysis spaces: Gene Ontology (Panel A), Cancer Modules (Panel B), standard differential genes (Panel C), and Gene Ontology terms derived from standard differentially expressed genes (Panel D). Each Venn diagram displays how well how each independent mechanism set overlaps in each of these spaces. The bold percentage in each panel provides the percentage of overlapping terms as a percentage of the average mechanism set length. Panel C demonstrates conventionally generated differentially expressed genes using the Significance Analysis of Microrarrays with a FDR of 5%. In panels A and B we first transform the gene expression arrays into either Biological Process Gene Ontology space or Cancer Module space, respectively. Individual pathway terms were analyzed standard t-test and adjusted for multiplicity. Terms with a FDR ≤ 5% were retained. The Gene Ontology terms in Panel D were generated by enriching the differentially expressed genes in Panel C using the DAVID tool and retaining terms with a FDR ≤ 5%. Legend: *Only concordantly deregulated mechanisms across datasets are counted in FAIME (e.g. significantly up-regulated ones in cancer against each other, then significantly down-regulated ones, then union of the two groups).

The first three datasets are used for mechanism feature selection reported in Table 2 and Figure 1. The fourth dataset serves as independent validation as depicted in Figure 3.

### Pathway-level datasets

To aggregate the genes into pathway-level mechanisms, we evaluated two different annotation repositories from two very differing etiologies: **Gene Ontology (GO) **and **Cancer Modules (CM) **[15]. Gene Ontology terms and their mappings to human genes for human genes were downloaded from National Center for Biotechnology Information at (ftp://ftp.ncbi.nlm.nih.gov/gene/DATA/gene2go.gz) on December 11, 2009. Cancer Modules were downloaded from Broad Institute (c4.cgn.v3.0.symbols.gmt on February 1, 2012).

GO relies on human curated annotations for determination of each pathway. In sharp contrast, Cancer Modules are a computationally derived set of pathways. The CM's are an integrated analysis of 1,975 published microarrays spanning 22 tumor types divided into modules. Each set of CM genes is derived from co-expression analyses that do not rely on curated knowledge or prior genesets.

Gene Ontology is organized into three separate ontologies. We chose to use the Biological Process subset as it had the appropriate scale of biology for pathway mechanisms. To avoid the oversampling bias introduced by multiple alternate probe-sets assigned to a gene, we used the probe-set of a gene with the largest coefficient of variation and discarded the others. By this approach, we intended to capture the gene most discriminative among diseases and controls and filtered the false signals in validation stage.

### Generating FAIME-derived mechanisms via gene sets

FAIME uses mRNA expression arrays as input and as output provides a score for each individual term for the gene annotation set (GO, CM) desired. The specifics of the algorithm have been described in detail previously[9]. For simplicity, FAIME-derived Gene Ontology mechanisms and Cancer Module mechanisms will be abbreviated FD-GO and FD-CM, respectively. In brief, for a given patient's gene expression profile, all expressed genes are sorted in a descending order according to the expression level. An exponential decreasing weight (**Eq. 1**) is then assigned to the ordered genes. The resultant weighted expression values are used to prioritize these highly expressed genes.

(1) $$w_{g,s}=\left(r_{g,s}\right)\cdot \left(e^{-\frac{r_{g,s}}{\left|G\right|}}\right)$$

**Eq. 1**. Weight *r_g,s _*assigned to gene *g *in total set of array genes *G *for a patient sample *s*, where *|G| *is the cardinality of *G*

(2a) $$\begin{array}{cc} \begin{array}{cc} NC{\left(GO_{i,s}\right)}_{}=\frac{1}{\left|GO_{i}\right|}\underset{g\in GO_{i}}{\sum }\left(w_{g,s}\right) & \end{array} & \end{array}$$

(2b) $$\begin{array}{cc} & NC\left(G/GO_{i,s}\right)=\frac{1}{\left|G/GO_{i}\right|}\underset{g\in G/GO_{i}}{\sum }\left(w_{g,s}\right) \end{array}$$

**where **$G/GO_{i}=\left\{g|g\notin GO_{i}\cap g\in G\right\}$

**Eq. 2a, 2b**. Normalized centroid *NC*(*GO_i,s_*), calculated within sample *s*, using weighted expression of genes associated to the Gene Ontology mechanism *i*. *G*/*GO_i, _*is the complement of *GO_i _*genes in the array gene set *G *.

(3a) $$FMraw\left(GO_{i,s}\right)=NC\left(GO_{i,s}\right)-NC\left(G/GO_{i,s}\right)$$

(3b) $$FM\left(GO_{i,s}\right)=FMraw\left(GO_{i,s}\right)-min_{j=1,2{,}_{}..._{}{,}_{}|M|}\left(FMraw\left(GO_{j,s}\right)\right)$$

**Eq. 3**. *FM(GO_i,s_) *is the FAIME score of Gene Ontology mechanism *i *in sample *s*. *M *is the set of mechanisms in GO; |*M*| is its cardinality. Cancer Module mechanisms are similarly calculated for each sample.

For each annotation term (based on its gene members), an average of the weighted expression values from the individual patient is calculated **(Eq. 2a)**. We then normalize this annotation term score (**Eq. 3a**) by subtracting the average of the complement set (genes NOT in the annotation term) **(Eq. 2b**). This process is repeated for every annotation term and for every patient. Finally, assuming the majority of pathways unchanged between sample groups, we run a within-sample normalization for these calculated individualized FAIME scores. FAIME scores of each sample are rescaled by using the minimum raw score of each sample (**Eq. 3b**).

### Derivation of FAIME tumorigenesis mechanisms (Table 2)

We compared individualized FAIME scores between two cohorts of patients (tumor versus normal) for a given dataset using a standard t-test. The resultant p-values were adjusted for multiplicity using the Benjamini false discovery rate (FDR) method. Terms with a FDR less than 5% and 1% were respectively retained as two distinct sets. We then carefully took note of the directionality of the between group change per term, according to the FAIME score per term. Specifically, a positive FAIME score indicates that the majority of the gene members of the measured pathway are up-regulated in tumor than in normal samples. Thus, our final FAIME-based tumorigenesis signature includes both an annotation term and its associated vector.

### Derivation of differentially expressed genes and Gene Ontology enrichment (Table 2)

To derive a list of differentially expressed genes from each of the datasets, we loaded the gene expression sets into R/Bioconductor and performed standard log normalization. We first filtered genes using a 2 fold-change cutoff, comparing tumor with normal samples. Highly differentially expressed genes were identified using Significance Analysis of Microarrays (SAM) algorithm in the Bioconductor *siggenes *package. SAM, by design, controls for multiplicity. Genes with an FDR of 5% and 1% were retained. We then performed Gene Ontology biological process pathway enrichment using the DAVID tool [16]. Finally, we kept GO terms that met a FDR of less than or equal to 5%.

### Determining pathway mechanism overlap across multiple datasets (Table 3)

To compute mechanism overlap among our FAIME-derived Cancer Module (FD-CM) and FAIME-derived Gene Ontology (FD-GO), we took advantage of the fact that FAIME mechanisms include both an annotation term and a direction of deregulation. Thus, a biologically meaningfully shared *tumorigenesis *mechanism between two signatures must be similarly matched on both (as differentially expressed FAIME scores can be either up- or down-regulated, concordance of the direction of the deregulation is required). To standardize the overlap comparison in Table 3, we provide the overlap in terms of percentage of each of the two original sets of deregulated mechanisms being compared.

### Determining significance of the FAIME-derived mechanism overlap across multiple datasets and for significance of overlap to known cancer genes

To compute the probability of FD-GO and FD-CM mechanistic overlap, we first converted the mechanisms to their constituent genes. We then used a bootstrap method of 10,000 draws of genes from the same FD-GO transformed background in R and from the FD-CM transformed background. We then computed the number of times the two sets overlapped at the desired target or higher. An empirical p-value was estimated for the significance of cross-dataset overlap. Similarly, we evaluated the genes overlapping between FD-GO and FD-CM with that of known cancer genes from the Wellcome Trust Sanger Institute Cancer Genome Project web site, http://www.sanger.ac.uk/genetics/CGP on March 3, 2012. We performed a bootstrap of 10,000 draws to generate an empiric p-value to evaluate whether observed the overlap between established cancer genes and the overlap could have occurred by chance alone. The R algorithm for Partitioning Around Medoids (PAM) was utilized in a parameter-free way to automatically generate two unbiased partitions from either the GO-terms or the "Cancer Modules" features associated to *tumorigenesis*.

## Results and discussion

### FAIME-derived mechanisms are exquisitely sensitive to phenotypic differences and provide greater coverage than differential gene expression analysis

We first examined the differences between prostate tumor and normal prostate. We used three independent datasets and identified patient groups of interest in each: Yu (109 samples), Wallace (69 samples), and Taylor (82 samples). We transformed the gene expression data into FAIME-space using the procedure outlined in the Methods. As with previous FAIME-derived mechanism generation, we noted tremendous heterogeneity among the datasets with regard to the highly variable number of significant mechanisms. The Yu dataset resulted in the largest sets of mechanisms (as a percentage of the search space) whereas the Wallace dataset consistently had the shortest. As would be expected with traditional differentially expressed gene sets generated using SAM at a FDR of 5% and 1%, they consistently represented only a small fraction of the original gene space from which they were derived (less than 5%) (Table 2).

**Table 2:** Comparison of FAIME-prioritized mechanisms against controls (standard bioinformatics)

*Human Prostate Cancer Dataset*	*FDR*	FAIME-Derived Mechanisms		Controls: Standard Bioinformatics Approaches*	
		*Gene Ontology (n = union of {4877 up-} ∪ {4877 down-} regulated)*	*Cancer Module (n = {454 up-} ∪ {454 down-} regulated)*	*Diff. Expressed Genes*	*GO Enrichment from Diff. Expressed Genes (# terms; n = 4877)*
**Yu**	0.01	**28% **(2703)	**25% **(230)	**2.3% **(208/8799)	**0.3% **(14)
	0.05	**32% **(3137)	**31% **(282)	**3.3% **(293/8799)	**0.4% **(21)
**Wallace**	0.01	**5% **(441)	**4% **(36)	**2.5% **(326/12680)	**0.1% **(4)
	0.05	**13% **(1226)	**9% **(83)	**4.5% **(574/12680)	**0.2% **(10)
**Taylor**	0.01	**17% **(1672)	**12% **(109)	**0.7% **(202/26448)	**0.1% **(6)
	0.05	**25% **(2395)	**19% **(168)	**1.0% **(266/26448)	**0.2% **(9)

*As a comparison, differentially expressed genes (SAM, FDR ≤ 0.05) were enriched for Gene Ontology (GO) Terms using the DAVID tool. Numbers in parentheses indicate significant terms and the universe of terms from which they were drawn to generate the percentage indicated.

As a point of comparison with our FD-GO mechanisms, we performed a standard bioinformatics Gene Ontology enrichment of the differentially expressed genes for each dataset. Interestingly, no terms were commonly identified by the conventional enrichment analyses (FDR cutoff of 5%). In contrast, FD-GO identified 13% to 48% overlap between each pair of datasets and 6% of overlap across all three datasets analyzed (Figure 1).

From this we concluded that FAIME-derived mechanisms are more reproducible among different experiments pertaining to the same disease. Further, FAIME-based methods provide an improvement in overall percentage coverage and sensitivity even in the same pathway space.

### Sets of FAIME-derived mechanisms overlap more than sets of differentially expressed genes

A primary characteristic of mRNA based differentially gene expression sets is their lack of consistency among datasets, patients and methods of generation. Indeed, when we explored the totality of differentially expressed genes at a FDR less than 5% in the three datasets, only 7 genes overlapped after taking into account shared directionality. Indeed, this pattern was repeated even when we examined the Gene Ontology mechanisms that were derived from direct enrichment of the differentially expressed sets of genes. No Gene Ontology terms overlapped in a meaningful manner (Figure 1, Table 3). In contrast, using FAIME, we use the totality of the expressed genes without "cherry-picking" them to develop mechanisms. To determine whether our FAIME-derived mechanisms were better able to detect common pathways between datasets we examined the percentage of genes that overlapped with the mean size of the significant mechanisms from which they were derived. At the overlap of all three datasets, were 14 FD-CM mechanisms (Supp Table 1 in Additional file 1), 137 FD-GO mechanisms (Supp Table 2 in Additional file 1), and conventional differentially expressed gene sets only overlapped by 7 genes (Supp Table 3 in Additional file 1). At face value, it may appear that the overlap of the differential expressed genes are meaningful, it is important to take into account the background from which they were derived - these overlap genes only represented about 1-3% of the size each set of differentially expressed genes. In comparison, the overlap FD-GO mechanisms overlapped three times more at 4-11% and the FD-CM mechanism overlapped even more so at 5-17%. In other words, despite dataset heterogeneity, FAIME-derived pathway mechanisms increased three- to four-fold the overlap in all the pairwise dataset comparisons. As an example, looking across Table 3 at the Yu/Taylor dataset overlap, we see FD-GO mechanisms overlapping at 30% and FD-CM mechanisms at 34%. The differentially expressed gene set overlapped at a mere 7%.

**Table 3:** Evaluation of the overlap among different approaches to prioritize classifier's mechanisms

Approaches to Prioritize Classifier's Mechanisms		*Overlapping Mechanism (#)*	*% of each compared set*	*Overlapping p-value (FET)*
**FAIME-Derived Approaches**	***Overlap-Gene Ontology***			
	Yu ∩ Wallace	462	15%; 38%	6.9 x10^-6^
	Yu ∩ Taylor	824	26%;34%	3.8 x10^-3^
	Wallace ∩ Taylor	360	29%;15%	2.2 x10^-5^
	Yu ∩ Wallace ∩ Taylor	137	4%;11%;6%	
	***Overlap Cancer Module***			
	Yu ∩ Wallace	41	15%;49%	1.9x10^-4^
	Yu ∩ Taylor	77	27%;46%	5.7 x10^-6^
	Wallace ∩ Taylor	25	30%;15%	4.8 x10^-4^
	Yu ∩ Wallace ∩ Taylor	14	5%;17%;8%	

**Controls: Standard Bioinformatics Approaches**	***Overlap of Differentially Expressed Genes***			
	Yu ∩ Wallace	33	11%;6%	6.1 x10^-9^
	Yu ∩ Taylor	65	22%;24%	<2 x10^-16^
	Wallace ∩ Taylor	20	3%;8%	1.5 x10^-3^
	Yu ∩ Wallace ∩ Taylor	7	2%;1%;3%	
	***Overlap GO derived from Differentially Expressed Genes***			
				
	Yu ∩ Wallace	1	5%;10%	4.2 x10^-2^
	Yu ∩ Taylor	0	NA	NS
	Wallace ∩ Taylor	0	NA	NS
	Yu ∩ Wallace ∩ Taylor	0	NA	NS

Legend: NA = not applicable; NS = not significant; #= count of significant mechanisms overlap

### FAIME-derived mechanisms recapitulate and reveal novel prostate cancer biology

Careful analysis of the overlap of the FAIME-derived mechanisms reveals pathways that are known players in tumorigenesis. For example, the top two pathways (smallest FDR value) that were increased in tumor relative to normal tissue in the FD-GO mechanistic overlap were GO:0040519 "negative regulation of nitric oxide biosynthetic process" and GO:007260 "tyrosine phosphorylation of STAT protein" (Supp Table 2 in Additional file 1). Interestingly, nitric oxide negatively regulates androgen receptor activity that is critical in all prostate cancer [17] and has been explored as a potential therapeutic modality. Similarly, activation of the STAT family of transcription factor by tyrosine kinases has been recognized informatically by ourselves and others as key drivers in prostate cancer [1,18].

Perhaps what is more interesting are the pathways that are unexpected or poorly described in the prostate cancer literature. Such an example may be GO:0019896 "axon transport of mitochondrion" which is hinted at by previous studies of a class of drugs that inhibit this pathway. Initial *in vitro *work has demonstrated anti-tumor effects in prostate cancer [19]. Although this potential biological drug class was identified, further pursuit of understanding the biological mechanisms underlying the role this pathway has not been further researched in prostate cancer.

Similarly, while the FD-CM mechanisms do not have formal associated annotations, they are informative as well. For example, Module 457 (Supp Table 1 in Additional file 1) is composed of 10 genes that are upregulated in prostate tumors as compared to normal tissue. Yet Gene Ontology enrichment in either the Biological Process or Molecular Function ontologies does not lead to statistically significant enrichment. Nevertheless, Module 457 contains the gene KPNA2 that has recently been identified as a potential biomarker for recurrent prostate disease [20]. KPNA2 is involved in nuclear transport and the other genes in the module, such as nucleolin, are part of the nuclear structure. The actual mechanism to its oncogenic activity still needs to be clarified. Thus a FAIME-derived Cancer Module approach allows us to identify new pathways that may not be fully elucidated or even discovered.

### Curated GO and computationally generated "Cancer Module" mechanisms overlap significantly and are enriched for known prostate cancer genes

**Figure 2:**
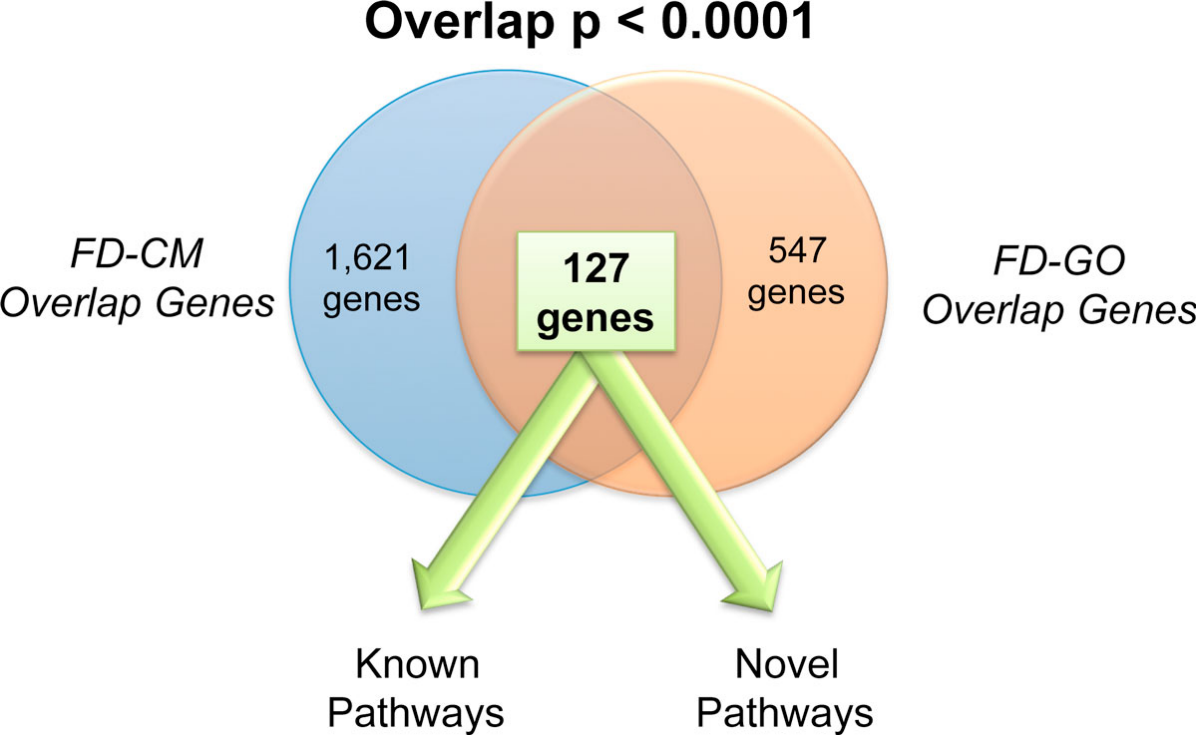
**FAIME-derived Gene Ontology and Cancer Module tumorigenesis mechanisms overlap significantly when we analyze their constituent genes by a bootstrap method to generate an empiric p-value**. 127 genes overlap between the two of them and are highly enriched for known and novel deregulated prostate cancer pathways.

After determining that, indeed, FAIME-derived mechanisms improve overlap among different independent datasets, we next explored whether these FAIME-derived mechanisms overlapped amongst themselves. In other words, do mechanisms from FD-GO and FD-CM overlap when we explore their shared common genes? To make this determination, we mapped each FAIME-derived overlap (the commonly shared mechanisms among all three datasets) back to their constituent genes and then examined their overlap. Figure 2 summarizes the results of our analysis. We determined that 127 genes were shared (empiric p-value: p < 0.0001) between the overlapping FD-CM mechanisms and those derived from the overlapping FD-GO mechanisms. We also examined if there were differentially expressed genes that were shared among the FD-GO and FD-CM mechanisms. Indeed, we observed that JUNB, a transcription factor that regulates growth response and has been implicated in prostate cancer progression [21] was in common among all three spaces.

We next proceeded to look more closely at the 127 genes shared by FD-GO and FD-CM (Supp Table 4 in Additional file 1). We performed two analyses. First, we asked whether or not these 127 genes were enriched for known cancer genes. Using the Sanger Cancer Gene Census, a curated list of known cancer genes (currently 474 genes), we determined that 14 genes overlapped significantly (empiric p-value < 0.0001) between our 127 genes and the Cancer Gene Census. This gave us further confidence that our method and the overlap genes were indeed highly related to tumorigenesis.

In our second analysis, we used the DAVID tool and determined the available annotations for this gene set. The Kyoto Encyclopedia of Genes and Genomes (KEGG) served as an independent source of pathway enrichment, two cancer-related KEGG terms came up as significant after correcting for multiplicity by Bonferonni, hsa05219: "bladder cancer" (*p *= 0.003) and hsa05200: "pathways in cancer" (*p *= 0.01). In addition, when we performed this enrichment using Gene Ontology, the top term in the Biological Process ontology was regulation of "cell proliferation" (Bonferonni: p < 0.0001). Again, this is inline with one of the key hallmarks of cancer. A complete list of the statistically significant terms is available in Supp Table 5 in Additional file 1.

### FAIME-derived mechanisms of tumorigenesis are predictive for prostate cancer recurrence

Because we generated our pathway mechanisms based on the difference between normal prostate and prostate cancer, we essentially developed a set of tumorigenesis mechanisms. We subsequently hypothesized that men with prostate cancer who developed recurrent disease would have greater deregulation of this tumorigenesis set. To test this hypothesis, we examined a well-annotated dataset of 79 men who underwent prostatectomy [14] and were followed until biochemical relapse (detectable prostate specific antigen). In these patients, PSA is a well-validated, powerful indicator predictor of prostate cancer progression (http://www.mskcc.org/cancer-care/adult/prostate/prediction-tools). In each of these cases, "pre-treatment", "post-prostatectomy", "salvage radiation" -- PSA values are critical for prediction of outcome. In patients who have had their entire prostate and associated cancer removed, the PSA should be undetectable. Thus, in later tests, if the PSA becomes detectable, this detection is termed "biochemical recurrence" of the prostate cancer.

**Figure 3:**
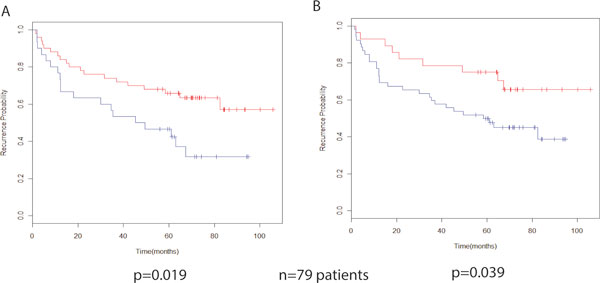
**Kaplan-Meier plots of recurrent prostate cancer using the FAIME-derived Cancer Module overlap set (Panel A) and the FAIME-derived Gene Ontology overlap set (Panel B)**. PAM analysis was used to divide patients into two cohorts. Statistically significant differences in time to recurrence are consistently observed based on log-rank statistic (*p *= 0.0186 and 0.0392, respectively).

We divided these men into two separate cohorts using the PAM algorithm using either the mechanisms from the FD-GO and the FD-CM overlaps. Kaplan-Meier analysis of the Glinsky dataset in Gene Ontology and Cancer Module space demonstrated a statistically different recurrence free interval (*p *= 0.039 and 0.018, respectively). Impressively, as seen in Figure 3, in the good prognosis cohort of the Kaplan-Meier curves, the 50% recurrence endpoint was not reached in either pathway mechanism set.

### Discussion

The relative overlap of mechanisms discovered between different prostate cancer cohorts appears modest, however the absolute overlap corresponds to 137 GO terms and 14 cancer modules. These somewhat small overlaps can be interpreted in the context of highly heterogeneous populations of prostate cancer patients. This dataset heterogeneity has also been reported as a fundamental challenge in reproducibility of gene signatures across solid tumor datasets and the FAIME methodology was nonetheless sensitive enough to significantly detect them. Here, we document at least four sources of heterogeneity as confounders in the results. First, Yu's samples are laser-microdissected and thus have no stromal expression whereas the Taylor dataset only required >70% cancer cells -as assessed by histology- thus allowing for ~30% stromal cells influence on the array expression. In a separate publication we have demonstrated that this change is profound and may mask or confound signal [22]. In the case of the Wallace dataset, we know that African-descent men comprise ~50% of the sample set. And indeed black men have a far higher rate of prostate cancer than European-descent men and are twice as likely as whites to die of the disease. Multiple publications (including Wallace et al.) have tried to define the set of genetic determinants that underlie this disparity. Unsurprisingly, FAIME detected a different set of enriched pathways than the other two (mostly European) sets - and this is the largest gap concordantly observed in term of GO as well as Cancer Modules mechanisms (Table 3). Furthermore, heterogeneity lies in dates and platforms for gene expression analysis. The Yu dataset published in 2004 uses different array technology than that of Taylor [13]. Although the techniques are similar, the reagents, probes, and chips have undergone a refinement process what amounts to over half a decade. In total, we, like the SAM, methodology are beholden to the underlying fundamentals of the datasets analyzed. Indeed, our previous work exploring non-overlapping gene expression signatures despite overlapping phenotypes [1] points to the need for more sensitive techniques, such as FAIME to be employed. Thus, the apparent decrease in specificity in mechanism detection prostate cancer as compared to that reported earlier in head and neck cancer [9], is most likely attributable to increased heterogeneity in prostate cancer datasets.

Computationally derived gene sets (e.g. "Cancer Modules") potentially lack one simple interpretation. Indeed, computationally-defined mechanisms may cross over or include co-expressed pathways that biologists may have envisioned as two different mechanisms. However, it is important to remember that this distinction was at a biologist's discretion or conceptualization. Computationally mechanisms that are unbiased may be closer to true transcriptome reality.

Another limitation is that the choice of size of the gene set to define a mechanism is in of itself open to debate. The determination of the statistical test used may also alter the results of a FAIME analysis. Taken together, the fundamental limitation of our methodology is that we are beholden to *a priori *defined mechanisms. The quality and context in which these gene sets/mechanisms are generated will ultimately affect our results.

Despite these limitations, computationally-generated gene sets still have the advantage of being generated in a principled, consistent, and unbiased manner. Researchers also have the ability to recreate computationally-generated sets as needed for differing situations, a process to which standard curated pathway mechanisms would not be amenable.

### Future directions

The combination of the FAIME algorithm using computationally defined mechanism is a natural fit for the deluge of next-generation sequencing data that is rapidly being generated. For example, RNA-seq technology identifies not only the level of transcription but also transcriptional variants (e.g. splice variants of the androgen receptor in prostate cancer) that may have differing phenotypic consequences. These gene variations are not accounted for in traditional pathway analysis using curated databases of mechanisms. In fact, it is unlikely there will be a human curated database in the short-term that will be able to maintain pace. Therefore, an agnostic, computational approach that is scalable, robust and reduces dimensionally in an efficient manner is essential.

We believe that our FAIME/computationally-defined mechanism paradigm will help address this. To this end, we are currently evaluating RNA-seq datasets in prostate cancer to test this hypothesis. By developing our own computational-defined mechanisms and then applying FAIME, we take advantage of all the patient prostate cancer data - not just explore genes or alleles with which we are familiar. Then by applying the FAIME algorithm, we can apply our mechanisms to individual patients - possibly for treatment decisions and/or improved risk stratification.

Finally, improved detection of unbiased genesets have recently been proposed and will systematically be investigated and contrasted against one another and the curated ones for utility in deriving classifiers of response to therapy: (i) differential interaction modules from Chen's group [23], (ii) dynamic transcriptional networks [24], (iii) genetic-expression interactions modules [25,26], etc.

## Conclusion

The extension of our FAIME methodology with curation-free biomodules ("Cancer Module") derived from the dynamic and congruent activation and/or deactivation of gene expression across numerous cancers breaks from the paradigm of recapitulating the known curated pathway mechanism universe. Furthermore, it empowers us to discover mechanistic deregulations that may be poorly characterized or even unknown. We demonstrate the utility and power of transforming gene expression arrays into unbiased pathway spaces comparable in accuracy to the FAIME-transformation using Gene Ontology that we had previously validated in head and neck and lung cancers. Pathway-derived mechanisms have the advantages of decreasing the overall dimensionality of the search space while increasing the sensitivity of detecting phenotypic differences. This allows for additional statistical power for exploring complex interactions of genes as compound biomarkers, as demonstrated by MammaPrint [27] and Oncotype DX [28] for breast cancers. As expected, we clearly demonstrate that mechanism-level overlap trumps gene-level overlap despite dataset heterogeneity, and this for both types of mechanisms: curated and curation-free ones. As mentioned, while they may lack clear functional classifications, computationally generated mechanisms, such as the Cancer Modules, recapitulate (i) known cancer genes, (ii) those predicted with curated GO pathways, and (iii) additionally provide coverage of genes that have yet to have a function assigned and would not have been discovered with the curated pathway paradigm.

Finally, in our application to prostate cancer, we demonstrate that either Gene Ontology or Cancer Module conceptualizations of a molecular pathway provide excellent discriminatory ability between normal prostate and tumor. These pathway mechanisms are of indisputable prognostic import when applied to independent clinical datasets. Because these pathway mechanisms can be applied at the individual patient level, a natural extension of this technology would be to develop customized predictors for recurrence and for survival. Thus, as the number and function of genes multiplies, pathway level mechanisms may ultimately become the *de facto *modality to interpret molecular deregulations in a convenient, quantitative and biologically anchored fashion.

## Competing interests

The authors have read and understood the BMC policy on declaration of interests and have no relevant interests to declare.

## Authors' contributions

The following authors have made substantial contributions to conception and design of the study (JLC, XY, YAL), the acquisition of data (JLC, AH, XY, JL, YL, GP, HL), the analysis and interpretation of data (JLC, AH, XY, JL, YL, HL, YAL), and the drafting or critical revising the manuscript for important intellectual content (JLC, AH, XY, JL, YL, HL, YAL). All authors read and approved the final manuscript.

## Acknowledgements

*Funding*: CTSA UL1RR029879, CTSA UL1RR024999, 3UL1RR024999-03S3, K22 LM008308-04, the University of Illinois Cancer Center, NIH-NCATS UL1TR000050, and the Cancer Research Foundation.

## Declarations

The publication costs for this article were funded by the corresponding author (YAL).

This article has been published as part of *BMC Medical Genomics *Volume 6 Supplement 2, 2013: Selected articles from the Second Annual Translational Bioinformatics Conference (TBC 2012). The full contents of the supplement are available online at http://www.biomedcentral.com/bmcmedgenomics/supplements/6/S2.

## Supplementary Material

Additional file 1 Click here for file

## References

[B1] Chen JL Li J Stadler WM Lussier YA Protein-network modeling of prostate cancer gene signatures reveals essential pathways in disease recurrence Journal of the American Medical Informatics Association 2011 18 4 392 402 10.1136/amiajnl-2011-000178 21672909 PMC3128407

[B2] Massagué J Sorting Out Breast-Cancer Gene Signatures New England Journal of Medicine 2007 356 3 294 297 10.1056/NEJMe068292 17229957

[B3] Sboner A Demichelis F Calza S Pawitan Y Setlur SR Hoshida Y Perner S Adami HO Fall K Mucci LA Molecular sampling of prostate cancer: a dilemma for predicting disease progression BMC medical genomics 2010 3 8 10.1186/1755-8794-3-8 20233430 PMC2855514

[B4] Lussier YA Stadler WM Chen JL Advantages of genomic complexity: bioinformatics opportunities in microRNA cancer signatures J Am Med Inform Assoc 2012 19 2 156 160 10.1136/amiajnl-2011-000419 22101905 PMC3277616

[B5] Chuang HY Lee E Liu YT Lee D Ideker T Network-based classification of breast cancer metastasis Mol Syst Biol 2007 3 140 17940530 10.1038/msb4100180 PMC2063581

[B6] Bild AH Yao G Chang JT Wang Q Potti A Chasse D Joshi MB Harpole D Lancaster JM Berchuck A Oncogenic pathway signatures in human cancers as a guide to targeted therapies Nature 2006 439 7074 353 357 10.1038/nature04296 16273092

[B7] Abraham G Kowalczyk A Loi S Haviv I Zobel J Prediction of breast cancer prognosis using gene set statistics provides signature stability and biological context BMC bioinformatics 2010 11 277 10.1186/1471-2105-11-277 20500821 PMC2895626

[B8] Iwamoto T Bianchini G Booser D Qi Y Coutant C Shiang CY Santarpia L Matsuoka J Hortobagyi GN Symmans WF Gene pathways associated with prognosis and chemotherapy sensitivity in molecular subtypes of breast cancer Journal of the National Cancer Institute 2011 103 3 264 272 10.1093/jnci/djq524 21191116

[B9] Yang X Regan K Huang Y Zhang Q Li J Seiwert TY Cohen EEW Xing HR Lussier YA Single Sample Expression-Anchored Mechanisms Predict Survival in Head and Neck Cancer PLoS Comput Biol 2012 8 1 e1002350 10.1371/journal.pcbi.1002350 22291585 PMC3266878

[B10] Subramanian A Tamayo P Mootha VK Mukherjee S Ebert BL Gillette MA Paulovich A Pomeroy SL Golub TR Lander ES Gene set enrichment analysis: A knowledge-based approach for interpreting genome-wide expression profiles Proceedings of the National Academy of Sciences of the United States of America 2005 102 43 15545 15550 10.1073/pnas.0506580102 16199517 PMC1239896

[B11] Yu YP Landsittel D Jing L Nelson J Ren B Liu L McDonald C Thomas R Dhir R Finkelstein S Gene expression alterations in prostate cancer predicting tumor aggression and preceding development of malignancy J Clin Oncol 2004 22 14 2790 2799 10.1200/JCO.2004.05.158 15254046

[B12] Wallace TA Prueitt RL Yi M Howe TM Gillespie JW Yfantis HG Stephens RM Caporaso NE Loffredo CA Ambs S Tumor immunobiological differences in prostate cancer between African-American and European-American men Cancer research 2008 68 3 927 936 10.1158/0008-5472.CAN-07-2608 18245496

[B13] Taylor BS Schultz N Hieronymus H Gopalan A Xiao Y Carver BS Arora VK Kaushik P Cerami E Reva B Integrative Genomic Profiling of Human Prostate Cancer Cancer Cell 2010 18 1 11 22 10.1016/j.ccr.2010.05.026 20579941 PMC3198787

[B14] Glinsky GV Glinskii AB Stephenson AJ Hoffman RM Gerald WL Gene expression profiling predicts clinical outcome of prostate cancer The Journal of clinical investigation 2004 113 6 913 923 15067324 10.1172/JCI20032 PMC362118

[B15] Segal E Friedman N Koller D Regev A A module map showing conditional activity of expression modules in cancer Nat Genet 2004 36 10 1090 1098 10.1038/ng1434 15448693 PMC2271138

[B16] Huang da W Sherman BT Lempicki RA Systematic and integrative analysis of large gene lists using DAVID bioinformatics resources Nat Protoc 2009 4 1 44 57 19131956 10.1038/nprot.2008.211

[B17] Siemens DR Heaton JP Adams MA Kawakami J Graham CH Phase II study of nitric oxide donor for men with increasing prostate-specific antigen level after surgery or radiotherapy for prostate cancer Urology 2009 74 4 878 883 10.1016/j.urology.2009.03.004 19476985

[B18] Abdulghani J Gu L Dagvadorj A Lutz J Leiby B Bonuccelli G Lisanti MP Zellweger T Alanen K Mirtti T Stat3 Promotes Metastatic Progression of Prostate Cancer The American Journal of Pathology 2008 172 6 1717 1728 10.2353/ajpath.2008.071054 18483213 PMC2408430

[B19] Sasse F Kunze B Gronewold TM Reichenbach H The chondramides: cytostatic agents from myxobacteria acting on the actin cytoskeleton Journal of the National Cancer Institute 1998 90 20 1559 1563 10.1093/jnci/90.20.1559 9790549

[B20] Mortezavi A Hermanns T Seifert HH Baumgartner MK Provenzano M Sulser T Burger M Montani M Ikenberg K Hofstadter F KPNA2 expression is an independent adverse predictor of biochemical recurrence after radical prostatectomy Clin Cancer Res 2011 17 5 1111 1121 10.1158/1078-0432.CCR-10-0081 21220479

[B21] Konishi N Shimada K Nakamura M Ishida E Ota I Tanaka N Fujimoto K Function of JunB in transient amplifying cell senescence and progression of human prostate cancer Clin Cancer Res 2008 14 14 4408 4416 10.1158/1078-0432.CCR-07-4120 18628455

[B22] Chen JL Li J Kiriluk KJ Rosen AM Paner GP Antic T Lussier YA Vander Griend DJ Deregulation of a Hox Protein Regulatory Network Spanning Prostate Cancer Initiation and Progression Clinical Cancer Research 2012 18 16 4291 4302 10.1158/1078-0432.CCR-12-0373 22723371 PMC3479663

[B23] Liu X Liu Z-P Zhao X-M Chen L Identifying disease genes and module biomarkers by differential interactions Journal of the American Medical Informatics Association 2012 19 2 241 248 10.1136/amiajnl-2011-000658 22190040 PMC3277635

[B24] Wang K Saito M Bisikirska BC Alvarez MJ Lim WK Rajbhandari P Shen Q Nemenman I Basso K Margolin AA Genome-wide identification of post-translational modulators of transcription factor activity in human B cells Nat Biotechnol 2009 27 9 829 839 10.1038/nbt.1563 19741643 PMC2753889

[B25] Gamazon ER Im H-K Duan S Lussier YA Cox NJ Dolan ME Zhang W ExprTarget: An Integrative Approach to Predicting Human MicroRNA Targets PLoS ONE 2010 5 10 e13534 10.1371/journal.pone.0013534 20975837 PMC2958831

[B26] Lee Y Yang X Huang Y Fan H Zhang Q Wu Y Li J Hasina R Cheng C Lingen MW Network Modeling Identifies Molecular Functions Targeted by miR-204 to Suppress Head and Neck Tumor Metastasis PLoS Comput Biol 2010 6 4 e1000730 10.1371/journal.pcbi.1000730 20369013 PMC2848541

[B27] van 't Veer LJ Dai H van de Vijver MJ He YD Hart AAM Mao M Peterse HL van der Kooy K Marton MJ Witteveen AT Gene expression profiling predicts clinical outcome of breast cancer Nature 2002 415 6871 530 536 10.1038/415530a 11823860

[B28] Paik S Shak S Tang G Kim C Baker J Cronin M Baehner FL Walker MG Watson D Park T A Multigene Assay to Predict Recurrence of Tamoxifen-Treated, Node-Negative Breast Cancer New England Journal of Medicine 2004 351 27 2817 2826 10.1056/NEJMoa041588 15591335

