# Supplementary material for: Curation-free biomodules mechanisms in prostate cancer predict recurrent disease
Source: BMC Med Genomics. 2013 May 7;6(Suppl 2):S4. doi: 10.1186/1755-8794-6-S2-S4 (PMC3654873; doi:10.1186/1755-8794-6-S2-S4)
Supplement: Additional file 1 [file 1755-8794-6-S2-S4-S1.pdf]

Supplemental Table 1: FAIME-Derived Cancer Module Overlap Among all 3 Sets of Prostate Cancer Gene Sets Evaluated

Cancer Modules	*Direction (Nml v Tumor)*	*P-value after FDR (Wallace)*	*P-value after FDR (Yu)*	*P-value after FDR (Taylor)*	*No. of Gene Members*
**module_16**	Down	0.003899	4.47E-13	0.000346	495
**module_103**	Up	0.022385	0.00682	0.040522	12
**module_123**	Down	0.024249	1.08E-05	0.001823	240
**module_126**	Down	0.000805	0.000129	1.84E-05	175
**module_139**	Down	0.013792	7.70E-05	0.015181	75
**module_164**	Up	0.003899	1.37E-05	0.048533	61
**module_197**	Down	0.000567	2.09E-12	0.00097	170
**module_236**	Down	0.013792	2.56E-30	0.000152	17
**module_260**	Down	0.013545	3.09E-30	0.028352	5
**module_264**	Down	0.017757	5.25E-16	0.004927	11
**module_287**	Up	0.007915	0.000561	0.000934	49
**module_400**	Up	0.010525	0.000259	0.00358	19
**module_408**	Down	0.003899	1.57E-19	0.000787	18
**module_457**	Down	0.003722	0.026199	0.000111	10

Supplemental Table 2: FAIME-Derived Gene Ontology Overlap Among all 3 Sets of Prostate Cancer Gene Sets Evaluated

Gene Ontology (Biological Process)	*Name*	*Direction (Nml vs. Cancer)*	*P-Value after FDR (Wallace)*	*P-Value after FDR (Yu)*	*P-Value after FDR (Taylor)*	*No. of Gene Members*
**GO:0051693**	actin filament capping	down	0.022172	0.005385	0.008958	17
**GO:0046086**	adenosine biosynthetic process	up	0.012849	1.41E-05	3.82E-05	1
**GO:0015866**	ADP transport	up	0.00181	1.02E-09	0.000199	1
**GO:0008344**	adult locomotory behavior	up	0.01469	1.22E-13	0.001611	28
**GO:0006196**	AMP catabolic process	up	0.011424	1.21E-06	0.02044	2
**GO:0006820**	anion transport	down	0.040654	8.64E-16	0.000168	18
**GO:0019369**	arachidonic acid metabolic process	up	0.013882	3.62E-08	7.04E-09	5
**GO:0015809**	arginine transport	down	0.042584	1.06E-17	9.99E-05	3
**GO:0015867**	ATP transport	up	0.00181	1.02E-09	0.000199	1
**GO:0019896**	axon transport of mitochondrion	up	0.016182	4.78E-05	0.00038	3
**GO:0016553**	base conversion or substitution editing	up	0.005335	0.000504	0.002477	3
**GO:0018874**	benzoate metabolic process	down	0.043171	0.003836	3.57E-08	1
**GO:0008206**	bile acid metabolic process	up	0.00398	1.46E-11	0.000745	12
**GO:0001824**	blastocyst development	up	0.037545	1.01E-07	1.01E-05	12
**GO:0030509**	BMP signaling pathway	up	0.01216	1.43E-06	0.020215	51
**GO:0019605**	butyrate metabolic process	down	0.043171	0.003836	3.57E-08	1
**GO:0060038**	cardiac muscle cell proliferation	up	0.0127	1.64E-12	0.000209	5
**GO:0070836**	caveola assembly	up	0.00616	5.39E-07	1.30E-11	2
**GO:0032488**	Cdc42 protein signal transduction	down	0.014864	7.79E-15	1.49E-05	4
**GO:0006875**	cellular metal ion homeostasis	down	0.029746	4.72E-26	0.001578	5
**GO:0034605**	cellular response to heat	up	0.009195	1.29E-12	2.27E-05	15
**GO:0031669**	cellular response to nutrient levels	up	0.003659	4.75E-07	1.23E-06	4
**GO:0007417**	central nervous system development	up	0.045984	3.07E-13	0.001427	93
**GO:0007099**	centriole replication	up	0.020635	2.72E-10	3.00E-05	6
**GO:0021895**	cerebral cortex neuron differentiation	up	0.040303	1.62E-05	4.58E-08	3
**GO:0006182**	cGMP biosynthetic process	up	0.004639	0.019006	0.004543	8
**GO:0060271**	cilium morphogenesis	up	0.008458	6.26E-08	0.000154	5
**GO:0051480**	cytosolic calcium ion homeostasis	up	0.014697	6.96E-12	1.61E-06	8
**GO:0060318**	definitive erythrocyte differentiation	up	0.039823	0.000342	0.000759	1
**GO:0050966**	detection of mechanical stimulus involved in sensory perception of pain	up	0.007798	2.53E-05	0.003677	4
**GO:0044111**	development involved in symbiotic interaction	up	0.007897	1.36E-05	0.0188	1

**GO:0060678**	dichotomous subdivision of terminal units involved in ureteric bud branching	down	0.011832	0.013867	0.000161	1
**GO:0006975**	DNA damage induced protein phosphorylation	down	0.007079	5.71E-17	3.37E-07	3
**GO:0042420**	dopamine catabolic process	down	0.019925	4.36E-13	0.028843	4
**GO:0007499**	ectoderm and mesoderm interaction	up	0.0127	1.48E-06	2.11E-11	2
**GO:0009880**	embryonic pattern specification	down	0.022543	0.036997	0.000659	12
**GO:0060136**	embryonic process involved in female pregnancy	down	0.012185	1.36E-07	0.011178	6
**GO:0040023**	establishment of nucleus localization	down	0.003868	3.05E-05	0.001355	3
**GO:0010994**	free ubiquitin chain polymerization	down	0.012571	3.07E-12	0.006613	2
**GO:0008347**	glial cell migration	up	0.011065	9.59E-09	0.000956	3
**GO:0032835**	glomerulus development	up	0.011709	0.004628	5.34E-07	4
**GO:0051935**	glutamate uptake involved in synaptic transmission	up	0.00181	1.02E-09	0.00852	2
**GO:0046168**	glycerol-3-phosphate catabolic process	up	0.026755	7.29E-07	5.90E-07	2
**GO:0034380**	high-density lipoprotein particle assembly	down	0.03745	2.28E-19	5.10E-05	5
**GO:0002418**	immune response to tumor cell	up	0.001217	0.000294	0.000644	1
**GO:0008629**	induction of apoptosis by intracellular signals	down	0.005751	0.002342	0.024593	14
**GO:0045110**	intermediate filament bundle assembly	up	0.004982	0.001368	7.49E-06	3
**GO:0006880**	intracellular sequestering of iron ion	up	0.007798	2.31E-09	0.034115	2
**GO:0043616**	keratinocyte proliferation	up	0.021349	4.98E-05	6.03E-12	10
**GO:0045324**	late endosome to vacuole transport	up	0.015432	4.29E-07	0.000288	1
**GO:0007159**	leukocyte cell-cell adhesion	down	0.024169	0.001526	0.011786	24
**GO:0042759**	long-chain fatty acid biosynthetic process	up	0.013713	1.39E-08	0.008243	2
**GO:0015909**	long-chain fatty acid transport	up	0.015695	0.015999	5.90E-07	5
**GO:0030098**	lymphocyte differentiation	down	0.002086	2.24E-12	0.046352	3
**GO:0048762**	mesenchymal cell differentiation	up	0.026714	2.68E-08	0.000228	5
**GO:0031109**	microtubule polymerization or depolymerization	up	0.021094	0.003815	4.29E-11	1
**GO:0030889**	negative regulation of B cell proliferation	down	0.034305	0.024881	0.002704	8
**GO:0090084**	negative regulation of inclusion body assembly	1.71E-05	0.034842	1.71E-05	0.011882	2
**GO:0032387**	negative regulation of intracellular transport	up	0.007927	0.003762	1.78E-08	2
**GO:0043409**	negative regulation of MAPKKK cascade	up	0.01778	1.64E-11	1.36E-10	8
**GO:0009892**	negative regulation of metabolic process	down	0.026516	2.65E-28	0.044318	2
**GO:0031441**	negative regulation of mRNA 3'-end processing	up	0.004855	0.012331	0.000252	1
**GO:0060547**	negative regulation of necrotic cell death	up	0.00181	1.02E-09	0.000199	1
**GO:0045019**	negative regulation of nitric	up	0.000112	1.07E-08	1.78E-05	5

	oxide biosynthetic process					
**GO:0010544**	negative regulation of platelet activation	down	0.036305	9.18E-17	0.000772	4
**GO:0048387**	negative regulation of retinoic acid receptor signaling pathway	down	0.014039	3.41E-21	5.36E-05	6
**GO:0045869**	negative regulation of retroviral genome replication	up	0.003937	0.014924	3.82E-05	2
**GO:0010766**	negative regulation of sodium ion transport	up	0.007897	1.36E-05	0.0188	1
**GO:0033144**	negative regulation of steroid hormone receptor signaling pathway	down	0.005262	2.04E-18	3.72E-05	2
**GO:0051964**	negative regulation of synaptogenesis	down	0.00359	3.05E-05	0.046765	3
**GO:0010768**	negative regulation of transcription from RNA polymerase II promoter in response to UV-induced DNA damage	up	0.007897	1.36E-05	0.0188	1
**GO:0042524**	negative regulation of tyrosine phosphorylation of Stat5 protein	up	0.005634	1.80E-07	5.97E-12	2
**GO:0048525**	negative regulation of viral reproduction	up	0.003937	0.014924	3.82E-05	2
**GO:0070050**	neuron homeostasis	up	0.046092	0.005791	0.002249	3
**GO:0001504**	neurotransmitter uptake	up	0.035267	0.024754	4.70E-10	4
**GO:0033484**	nitric oxide homeostasis	up	0.02432	4.70E-07	4.05E-12	1
**GO:0001866**	NK T cell proliferation	up	0.005463	0.000309	1.34E-06	2
**GO:0045292**	nuclear mRNA cis splicing, via spliceosome	up	0.004889	5.75E-09	0.011216	1
**GO:0009166**	nucleotide catabolic process	up	0.003892	1.66E-07	0.000112	3
**GO:0009225**	nucleotide-sugar metabolic process	down	0.014599	0.046995	0.008774	1
**GO:0001541**	ovarian follicle development	up	0.002243	1.19E-05	2.02E-09	33
**GO:0001542**	ovulation from ovarian follicle	up	0.018384	0.001124	0.003825	9
**GO:0006800**	oxygen and reactive oxygen species metabolic process	up	0.00836	0.000109	1.59E-08	24
**GO:0002502**	peptide antigen assembly with MHC class I protein complex	down	0.003937	2.75E-18	6.11E-05	1
**GO:0007422**	peripheral nervous system development	up	0.026654	0.001377	0.000585	23
**GO:0042986**	positive regulation of amyloid precursor protein biosynthetic process	down	0.025157	4.78E-19	0.008715	2
**GO:0045722**	positive regulation of gluconeogenesis	up	0.007798	4.93E-05	1.91E-06	2
**GO:0010838**	positive regulation of keratinocyte proliferation	up	0.032477	2.07E-05	5.88E-05	2
**GO:0010592**	positive regulation of lamellipodium assembly	down	0.043695	2.05E-08	0.002768	3
**GO:0002053**	positive regulation of mesenchymal cell proliferation	up	0.021082	0.000726	2.64E-06	15
**GO:0046824**	positive regulation of nucleocytoplasmic transport	up	0.007897	1.36E-05	0.0188	1
**GO:0051281**	positive regulation of release of sequestered calcium ion into cytosol	up	0.012371	1.79E-06	0.000594	9
**GO:0035025**	positive regulation of Rho	down	0.003277	3.87E-20	0.000224	4

	protein signal transduction					
**GO:0051973**	positive regulation of telomerase activity	up	0.00836	5.16E-11	8.43E-07	4
**GO:0051971**	positive regulation of transmission of nerve impulse	up	0.021797	0.003956	0.00077	3
**GO:0031503**	protein complex localization	down	0.002101	1.23E-05	0.007122	4
**GO:0070207**	protein homotrimerization	down	0.004124	2.30E-27	0.000382	6
**GO:0006493**	protein O-linked glycosylation	down	0.007798	7.43E-06	8.31E-06	17
**GO:0030091**	protein repair	up	0.002101	1.84E-09	1.38E-07	4
**GO:0000042**	protein targeting to Golgi	up	0.014839	3.20E-09	0.000548	2
**GO:0009956**	radial pattern formation	up	0.014186	9.29E-05	0.004155	1
**GO:0051481**	reduction of cytosolic calcium ion concentration	up	0.00181	1.70E-10	2.28E-05	4
**GO:0008064**	regulation of actin polymerization or depolymerization	up	0.009237	9.23E-07	0.005061	3
**GO:0045767**	regulation of anti-apoptosis	down	0.024848	1.48E-27	0.000198	2
**GO:0032012**	regulation of ARF protein signal transduction	down	0.006791	1.66E-21	0.011084	16
**GO:0005981**	regulation of glycogen catabolic process	up	0.019457	0.049745	6.03E-12	2
**GO:0019747**	regulation of isoprenoid metabolic process	up	0.034729	4.13E-06	0.001315	1
**GO:0045667**	regulation of osteoblast differentiation	up	0.016555	5.65E-09	0.000402	5
**GO:0052547**	regulation of peptidase activity	up	0.02432	4.70E-07	4.05E-12	1
**GO:0035020**	regulation of Rac protein signal transduction	down	0.029015	9.63E-33	0.000117	3
**GO:0032318**	regulation of Ras GTPase activity	down	0.003529	7.97E-06	1.43E-08	1
**GO:0006940**	regulation of smooth muscle contraction	up	0.004397	9.60E-07	4.00E-10	11
**GO:0050804**	regulation of synaptic transmission	up	0.007318	0.004494	0.003278	9
**GO:0060849**	regulation of transcription involved in lymphatic endothelial cell fate commitment	up	0.004526	6.38E-05	0.009798	2
**GO:0022904**	respiratory electron transport chain	up	0.00808	2.41E-05	0.009426	5
**GO:0032354**	response to follicle-stimulating hormone stimulus	up	0.039823	0.000342	0.000759	1
**GO:0010040**	response to iron(II) ion	up	0.02368	1.56E-11	0.008535	2
**GO:0010226**	response to lithium ion	down	0.014186	8.68E-15	0.00898	5
**GO:0034699**	response to luteinizing hormone stimulus	up	0.039823	0.000342	0.000759	1
**GO:0033574**	response to testosterone stimulus	up	0.014839	0.000247	0.0001	9
**GO:0042573**	retinoic acid metabolic process	up	0.016149	8.46E-11	3.77E-13	7
**GO:0001523**	retinoid metabolic process	up	0.006493	3.71E-12	1.93E-06	3
**GO:0010149**	senescence	down	0.014082	2.47E-18	0.000171	14
**GO:0051208**	sequestering of calcium ion	down	0.004287	2.89E-09	1.83E-08	3
**GO:0008295**	spermidine biosynthetic process	down	0.014661	4.21E-23	0.001992	3
**GO:0002223**	stimulatory C-type lectin receptor signaling pathway	up	0.005634	0.000148	0.000872	2

**GO:0051403**	stress-activated MAPK cascade	up	0.005047	0.040169	1.45E-10	3
**GO:0006410**	transcription, RNA-dependent	up	0.003937	0.014924	3.82E-05	2
**GO:0019089**	transmission of virus	up	0.007897	1.36E-05	0.0188	1
**GO:0001829**	trophectodermal cell differentiation	down	0.014186	1.25E-06	0.001165	11
**GO:0007260**	tyrosine phosphorylation of STAT protein	down	0.000764	5.23E-34	3.95E-05	3
**GO:0015785**	UDP-galactose transport	down	0.004133	1.66E-09	0.000733	1
**GO:0006212**	uracil catabolic process	up	0.00359	1.56E-06	1.21E-05	3
**GO:0046502**	uroporphyrinogen III metabolic process	up	0.001804	6.51E-06	0.039428	1
**GO:0007033**	vacuole organization	up	0.009656	8.49E-08	0.041288	3
**GO:0042310**	vasoconstriction	up	0.034824	1.78E-08	8.60E-08	3
**GO:0016050**	vesicle organization	up	0.014517	5.42E-09	0.000466	6

Supplemental Table 3: Overlap of Differentially Expressed Prostate Cancer Genes in 3 Prostate Cancer Datasets (Criteria: FDR < 5% in their individual datasets by SAM)

Symbol	***Name***	***Direction***
**SLC4A4**	solute carrier family 4, sodium bicarbonate cotransporter, member 4	UP
**AMACR**	alpha-methylacyl-CoA racemase	UP
**CXCR4**	chemokine (C-X-C motif) receptor 4	UP
**KLF6**	Kruppel-like factor 6	UP
**JUNB**	jun B proto-oncogene	UP
**SLC2A3**	solute carrier family 2 (facilitated glucose transporter), member 3	UP
**CRISP3**	cysteine-rich secretory protein 3	UP

Supplemental Table 4: FAIME-CM and FAIME-GO Gene Overlap
(Genes in red are known cancer related genes in the Wellcome Trust Sanger Cancer Gene Census)

Symbol	*Name*	*Symbol*	*Name*
**ACD**	adrenocortical dysplasia homolog (mouse)	**JUNB**	jun B proto-oncogene
**AGT**	angiotensinogen (serpin peptidase inhibitor, clade A, member 8)	**KDR**	kinase insert domain receptor (a type III receptor tyrosine kinase)
**AKR1C1**	aldo-keto reductase family 1, member C1 (dihydrodiol dehydrogenase 1; 20-alpha (3-alpha)-hydroxysteroid dehydrogenase)	**LDLR**	low density lipoprotein receptor
**ALDH1A2**	aldehyde dehydrogenase 1 family, member A2	**MAPK12**	mitogen-activated protein kinase 12
**ALDH1A3**	aldehyde dehydrogenase 1 family, member A3	**MIF**	macrophage migration inhibitory factor (glycosylation-inhibiting factor)
**ALDH3A2**	aldehyde dehydrogenase 3 family, member A2	**MSX1**	msh homeobox 1
**ALOX5AP**	arachidonate 5-lipoxygenase-activating protein	**MSX2**	msh homeobox 2
**ANGPT1**	angiopoietin 1	**NDN**	necdin homolog (mouse)
**APOA1**	apolipoprotein A-I	**NELL1**	NEL-like 1 (chicken)
**APOE**	apolipoprotein E	**NOX4**	NADPH oxidase 4
**ARSA**	arylsulfatase A	**NPAS1**	neuronal PAS domain protein 1
**ARSB**	arylsulfatase B	**NPC1**	Niemann-Pick disease, type C1
**BAAT**	bile acid CoA: amino acid N-acyltransferase (glycine N-choloyltransferase)	**NPTX1**	neuronal pentraxin I
**BCL2L1**	BCL2-like 1	**NPY1R**	neuropeptide Y receptor Y1
**BDKRB1**	bradykinin receptor B1	**NR4A2**	nuclear receptor subfamily 4, group A, member 2
**BIRC5**	baculoviral IAP repeat containing 5	**NTSR1**	neurotensin receptor 1 (high affinity)
**BMP10**	bone morphogenetic protein 10	**OPA1**	optic atrophy 1 (autosomal dominant)
**BMP7**	bone morphogenetic protein 7	**PAWR**	PRKC, apoptosis, WT1, regulator

**BNIP3**	BCL2/adenovirus E1B 19kDa interacting protein 3		**PAX6**	paired box 6
**CALR**	calreticulin		**PCP4**	Purkinje cell protein 4
**CARTPT**	CART prepropeptide		**PDGFB**	platelet-derived growth factor beta polypeptide
**CAV1**	caveolin 1, caveolae protein, 22kDa		**PEBP1**	phosphatidylethanolamine binding protein 1
**CCND2**	cyclin D2		**PPARG**	peroxisome proliferator-activated receptor gamma
**CDH1**	cadherin 1, type 1, E-cadherin (epithelial)		**PPP3CA**	protein phosphatase 3, catalytic subunit, alpha isozyme
**CDKN1A**	cyclin-dependent kinase inhibitor 1A (p21, Cip1)		**PRAME**	preferentially expressed antigen in melanoma
**CDKN2A**	cyclin-dependent kinase inhibitor 2A		**PRKCD**	protein kinase C, delta
**CITED2**	Cbp/p300-interacting transactivator, with Glu/Asp-rich carboxy-terminal domain, 2		**PRKDC**	protein kinase, DNA-activated, catalytic polypeptide
**CLN3**	ceroid-lipofuscinosis, neuronal 3		**PROP1**	PROP paired-like homeobox 1
**CRABP2**	cellular retinoic acid binding protein 2		**PRRX2**	paired related homeobox 2
**CRK**	v-crk sarcoma virus CT10 oncogene homolog (avian)		**PSD4**	pleckstrin and Sec7 domain containing 4
**CRYAA**	crystallin, alpha A		**PSMC4**	proteasome (prosome, macropain) 26S subunit, ATPase, 4
**CRYAB**	crystallin, alpha B		**PTK2B**	PTK2B protein tyrosine kinase 2 beta
**CSTB**	cystatin B (stefin B)		**PTPRC**	protein tyrosine phosphatase, receptor type, C
**CUL4A**	cullin 4A		**RBP1**	retinol binding protein 1, cellular
**CYP2D6**	cytochrome P450, family 2, subfamily D, polypeptide 6		**RCAN1**	regulator of calcineurin 1
**DHX9**	DEAH (Asp-Glu-Ala-His) box polypeptide 9		**RCAN2**	regulator of calcineurin 2
**EDNRB**	endothelin receptor type B		**RDX**	radixin
**ELF4**	E74-like factor 4 (ets domain transcription factor)		**SDHB**	succinate dehydrogenase complex, subunit B, iron sulfur (Ip)

**ESR2**	estrogen receptor 2 (ER beta)		**SERBP1**	SERPINE1 mRNA binding protein 1
**ESRRA**	estrogen-related receptor alpha		**SFN**	stratifin
**EZH2**	enhancer of zeste homolog 2 (Drosophila)		**SIM2**	single-minded homolog 2 (Drosophila)
**FADS1**	fatty acid desaturase 1		**SLC1A3**	solute carrier family 1 (glial high affinity glutamate transporter), member 3
**FBXO5**	F-box protein 5		**SLC4A10**	solute carrier family 4, sodium bicarbonate transporter, member 10
**FGF1**	fibroblast growth factor 1 (acidic)		**SLC4A2**	solute carrier family 4, anion exchanger, member 2 (erythrocyte membrane protein band 3-like 1)
**FLT3**	fms-related tyrosine kinase 3		**SMAD1**	SMAD family member 1
**GAS1**	growth arrest-specific 1		**SMAD5**	SMAD family member 5
**GLA**	galactosidase, alpha		**SNCG**	synuclein, gamma (breast cancer-specific protein 1)
**GMDS**	GDP-mannose 4,6-dehydratase		**SOX10**	SRY (sex determining region Y)-box 10
**GRIK1**	glutamate receptor, ionotropic, kainate 1		**STAT1**	signal transducer and activator of transcription 1, 91kDa
**GUCY2D**	guanylate cyclase 2D, membrane (retina-specific)		**TAF4**	TAF4 RNA polymerase II, TATA box binding protein (TBP)-associated factor, 135kDa
**GUCY2F**	guanylate cyclase 2F, retinal		**TBCE**	tubulin folding cofactor E
**HDAC2**	histone deacetylase 2		**THBS1**	thrombospondin 1
**HNF1A**	HNF1 homeobox A		**TIMP3**	TIMP metallopeptidase inhibitor 3
**HRAS**	v-Ha-ras Harvey rat sarcoma viral oncogene homolog		**TIMP4**	TIMP metallopeptidase inhibitor 4
**HSF1**	heat shock transcription factor 1		**TLX1**	T-cell leukemia homeobox 1
**HSF4**	heat shock transcription factor 4		**TMSB4Y**	thymosin beta 4, Y-linked
**ID4**	inhibitor of DNA binding 4, dominant negative helix-loop-helix protein		**TOMM40**	translocase of outer mitochondrial membrane 40 homolog (yeast)
**IGFBP2**	insulin-like growth factor binding protein 2, 36kDa		**TP53**	tumor protein p53

**IL6ST**	interleukin 6 signal transducer (gp130, oncostatin M receptor)		**UBE2C**	ubiquitin-conjugating enzyme E2C
**IMPA1**	inositol(myo)-1(or 4)-monophosphatase 1		**UGT8**	UDP glycosyltransferase 8
**INPP5D**	inositol polyphosphate-5-phosphatase, 145kDa		**VDAC2**	voltage-dependent anion channel 2
**IRF6**	interferon regulatory factor 6		**VEGFA**	vascular endothelial growth factor A
**ISL1**	ISL LIM homeobox 1		**ZEB2**	zinc finger E-box binding homeobox 2
**ITGB2**	integrin, beta 2 (complement component 3 receptor 3 and 4 subunit)			

Supplemental Table 5: Gene Ontology Enrichment of FD-GO and FD-CM Overlap Genes

Term	*Bonferroni adjusted p-value*
**GO:0042127~regulation of cell proliferation**	6.15E-22
**GO:0010033~response to organic substance**	7.12E-15
**GO:0009719~response to endogenous stimulus**	9.74E-14
**GO:0042981~regulation of apoptosis**	2.49E-13
**GO:0010604~positive regulation of macromolecule metabolic process**	2.49E-13
**GO:0043067~regulation of programmed cell death**	2.49E-13
**GO:0010941~regulation of cell death**	4.98E-13
**GO:0008285~negative regulation of cell proliferation**	6.22E-12
**GO:0009725~response to hormone stimulus**	9.21E-12
**GO:0048545~response to steroid hormone stimulus**	2.99E-11
**GO:0009628~response to abiotic stimulus**	9.08E-11
**GO:0008284~positive regulation of cell proliferation**	1.19E-09
**GO:0010605~negative regulation of macromolecule metabolic process**	1.46E-09
**GO:0031328~positive regulation of cellular biosynthetic process**	1.47E-09
**GO:0043627~response to estrogen stimulus**	1.78E-09
**GO:0009891~positive regulation of biosynthetic process**	2.13E-09
**GO:0032268~regulation of cellular protein metabolic process**	3.00E-09
**GO:0051173~positive regulation of nitrogen compound metabolic process**	1.19E-08
**GO:0048878~chemical homeostasis**	1.63E-08
**GO:0010557~positive regulation of macromolecule biosynthetic process**	1.72E-08
**GO:0043066~negative regulation of apoptosis**	2.59E-08
**GO:0043069~negative regulation of programmed cell death**	3.38E-08
**GO:0045935~positive regulation of nucleobase, nucleoside, nucleotide and nucleic acid metabolic process**	3.42E-08
**GO:0060548~negative regulation of cell death**	3.56E-08
**GO:0006357~regulation of transcription from RNA polymerase II promoter**	3.81E-08

**GO:0031399~regulation of protein modification process**	6.63E-08
**GO:0042592~homeostatic process**	8.36E-08
**GO:0001932~regulation of protein amino acid phosphorylation**	1.46E-07
**GO:0009991~response to extracellular stimulus**	4.57E-07
**GO:0045944~positive regulation of transcription from RNA polymerase II promoter**	4.66E-07
**GO:0044092~negative regulation of molecular function**	5.59E-07
**GO:0051174~regulation of phosphorus metabolic process**	1.42E-06
**GO:0019220~regulation of phosphate metabolic process**	1.42E-06
**GO:0043086~negative regulation of catalytic activity**	1.68E-06
**GO:0045767~regulation of anti-apoptosis**	3.93E-06
**GO:0042325~regulation of phosphorylation**	4.12E-06
**GO:0031667~response to nutrient levels**	8.99E-06
**GO:0007568~aging**	1.29E-05
**GO:0045768~positive regulation of anti-apoptosis**	1.96E-05
**GO:0010648~negative regulation of cell communication**	2.19E-05
**GO:0045941~positive regulation of transcription**	2.31E-05
**GO:0042493~response to drug**	2.93E-05
**GO:0045893~positive regulation of transcription, DNA-dependent**	3.55E-05
**GO:0010628~positive regulation of gene expression**	3.95E-05
**GO:0051254~positive regulation of RNA metabolic process**	4.08E-05
**GO:0051252~regulation of RNA metabolic process**	5.41E-05
**GO:0010035~response to inorganic substance**	1.29E-04
**GO:0010647~positive regulation of cell communication**	1.48E-04
**GO:0043085~positive regulation of catalytic activity**	1.48E-04
**GO:0007584~response to nutrient**	1.62E-04
**GO:0043065~positive regulation of apoptosis**	2.05E-04
**GO:0043068~positive regulation of programmed cell death**	2.28E-04
**GO:0045596~negative regulation of cell differentiation**	2.39E-04

**GO:0010942~positive regulation of cell death**	2.44E-04
**GO:0032269~negative regulation of cellular protein metabolic process**	2.49E-04
**GO:0007167~enzyme linked receptor protein signaling pathway**	2.52E-04
**GO:0006355~regulation of transcription, DNA-dependent**	2.91E-04
**GO:0003006~reproductive developmental process**	3.30E-04
**GO:0051248~negative regulation of protein metabolic process**	3.78E-04
**GO:0014070~response to organic cyclic substance**	4.06E-04
**GO:0032535~regulation of cellular component size**	4.99E-04
**GO:0035270~endocrine system development**	5.27E-04
**GO:0051260~protein homooligomerization**	5.31E-04
**GO:0050801~ion homeostasis**	5.34E-04
**GO:0001558~regulation of cell growth**	5.64E-04
**GO:0032270~positive regulation of cellular protein metabolic process**	5.75E-04
**GO:0032101~regulation of response to external stimulus**	5.95E-04
**GO:0009890~negative regulation of biosynthetic process**	7.10E-04
**GO:0051241~negative regulation of multicellular organismal process**	8.13E-04
**GO:0051247~positive regulation of protein metabolic process**	9.32E-04
**GO:0051726~regulation of cell cycle**	9.73E-04
**GO:0043009~chordate embryonic development**	9.73E-04
**GO:0044093~positive regulation of molecular function**	0.001013949
**GO:0055082~cellular chemical homeostasis**	0.001048719
**GO:0008361~regulation of cell size**	0.00107596
**GO:0009792~embryonic development ending in birth or egg hatching**	0.001091371
**GO:0030334~regulation of cell migration**	0.001099007
**GO:0048732~gland development**	0.001132476
**GO:0040008~regulation of growth**	0.001419534
**GO:0051259~protein oligomerization**	0.001470353
**GO:0045165~cell fate commitment**	0.001484339
**GO:0070482~response to oxygen levels**	0.00169364

**GO:0009612~response to mechanical stimulus**	0.001828424
**GO:0009266~response to temperature stimulus**	0.002228892
**GO:0009968~negative regulation of signal transduction**	0.002272758
**GO:0031327~negative regulation of cellular biosynthetic process**	0.00228163
**GO:0030301~cholesterol transport**	0.002379526
**GO:0015918~sterol transport**	0.002379526
**GO:0044057~regulation of system process**	0.002427053
**GO:0008015~blood circulation**	0.002845044
**GO:0003013~circulatory system process**	0.002845044
**GO:0007610~behavior**	0.003581207
**GO:0040012~regulation of locomotion**	0.003883976
**GO:0016477~cell migration**	0.003912288
**GO:0051270~regulation of cell motion**	0.004086148
**GO:0010243~response to organic nitrogen**	0.004128153
**GO:0051338~regulation of transferase activity**	0.004212561
**GO:0006873~cellular ion homeostasis**	0.004500818
**GO:0030308~negative regulation of cell growth**	0.004898288
**GO:0055080~cation homeostasis**	0.005798702
**GO:0032504~multicellular organism reproduction**	0.005962139
**GO:0048609~reproductive process in a multicellular organism**	0.005962139
**GO:0042698~ovulation cycle**	0.006285081
**GO:0010594~regulation of endothelial cell migration**	0.006615291
**GO:0001568~blood vessel development**	0.006672327
**GO:0055065~metal ion homeostasis**	0.00733545
**GO:0007242~intracellular signaling cascade**	0.007387679
**GO:0042326~negative regulation of phosphorylation**	0.007823085
**GO:0009967~positive regulation of signal transduction**	0.008143592
**GO:0045792~negative regulation of cell size**	0.008517288
**GO:0001944~vasculature development**	0.008558646

**GO:0048585~negative regulation of response to stimulus**	0.009183269
**GO:0050678~regulation of epithelial cell proliferation**	0.009305644
**GO:0010629~negative regulation of gene expression**	0.00943146
**GO:0001666~response to hypoxia**	0.009675128
**GO:0048514~blood vessel morphogenesis**	0.009676839
**GO:0007050~cell cycle arrest**	0.011450237
**GO:0010563~negative regulation of phosphorus metabolic process**	0.011499239
**GO:0045936~negative regulation of phosphate metabolic process**	0.011499239
**GO:0021983~pituitary gland development**	0.01193415
**GO:0050865~regulation of cell activation**	0.011947694
**GO:0051674~localization of cell**	0.012551804
**GO:0048870~cell motility**	0.012551804
**GO:0051172~negative regulation of nitrogen compound metabolic process**	0.013891841
**GO:0019725~cellular homeostasis**	0.014776875
**GO:0043010~camera-type eye development**	0.015188785
**GO:0007389~pattern specification process**	0.016045796
**GO:0055088~lipid homeostasis**	0.016454263
**GO:0045926~negative regulation of growth**	0.018620077
**GO:0055074~calcium ion homeostasis**	0.022387984
**GO:0032355~response to estradiol stimulus**	0.022988497
**GO:0051094~positive regulation of developmental process**	0.024037855
**GO:0031644~regulation of neurological system process**	0.02822205
**GO:0009408~response to heat**	0.028385636
**GO:0006875~cellular metal ion homeostasis**	0.032066981
**GO:0031400~negative regulation of protein modification process**	0.032995767
**GO:0052547~regulation of peptidase activity**	0.033096159
**GO:0035113~embryonic appendage morphogenesis**	0.035669082
**GO:0030326~embryonic limb morphogenesis**	0.035669082

**GO:0051240~positive regulation of multicellular organismal process**	0.03777147
**GO:0006461~protein complex assembly**	0.039705592
**GO:0070271~protein complex biogenesis**	0.039705592
**GO:0030335~positive regulation of cell migration**	0.041297553
**GO:0007422~peripheral nervous system development**	0.042460315
**GO:0021536~diencephalon development**	0.048575866
